# Single Stage Surgical Outcomes for Large Angle Intermittent Exotropia

**DOI:** 10.1371/journal.pone.0150508

**Published:** 2016-02-26

**Authors:** Min Yang, Jingchang Chen, Tao Shen, Ying Kang, Daming Deng, Xiaoming Lin, Heping Wu, Qiwen Chen, Xuelian Ye, Jianqun Li, Jianhua Yan

**Affiliations:** 1 Department of strabismus and amblyopia, The State Key Laboratory of Ophthalmology, Zhongshan Ophthalmic Center, Sun Yat-sen University, Guangzhou, Guangdong, the People’s Republic of China; 2 Department of strabismus and amblyopia, Tongde Hospital of Zhejiang Province, Hangzhou, Zhejiang, the People’s Republic of China; Bascom Palmer Eye Institute, University of Miami School of Medicine, UNITED STATES

## Abstract

Although there were many prior studies about exotropia, few focused on large-angle intermittent exotropia. The goal of this study was to evaluate single-stage surgical outcomes for large-angle intermittent exotropia and analyze risk factors that may affect the success of surgery. Records from intermittent exotropia patients with exodeviations >60 prism diopters(PD) who were surgically treated at the Zhongshan Ophthalmic Center, of Sun Yat-Sen University were reviewed. Included within this review were data on, pre- and post-operative ocular motility, primary alignment, binocular vision and complications. Patients with exodeviations ≤70PD received two-muscle surgery, while those with exodeviations >70PD were subjected to a three-muscle procedure. A total of 40 records were reviewed. The mean exodeviation was 73±9PD at distance and 75±26PD at near. There were 25 patients received two-muscle surgery and 15 the three-muscle procedure. Orthophoria (deviation within 8PD) was obtained in 77.5% of these patients and the ratios of surgical under-correction and over-correction were 15% and 7.5% respectively. However, when combining ocular alignment with binocular vision as the success criteria, success rates decreased to 30%. No statistically significant differences in success rates were obtained between the two- and three-muscle surgery groups. Seven subjects experienced an abduction deficit during the initial postoperative stages, but eventually showed a full recovery. One patient required a second surgery for overcorrection. No statistically significant risk factors for poor outcome were revealed. Our data showed that single-stage two- and three-muscle surgeries for large-angle intermittent exotropia are effective in achieving a favorable outcome.

## Introduction

Intermittent exotropia (IXT) represents the most common type of exotropia, with an incidence of 32.1 per 100,000 in adolescents [1]. While the surgical treatment of IXT has been described within a number of reports, few have focused on large-angle IXT; and, previous studies involved with large-angle strabismus have concentrated on all exotropic cases including both IXT and constant exotropia. Recommended surgical doses for this condition were designated as up to 50PD[2], however many issues regarding large-angle IXT remain unresolved. The first being the definition of large-angle, with values in the literature ranging from 40 to 60 PD [3–5]. Second, is that of the variations in surgical managements, where two-, three- or four-muscle surgeries have been recommended. Third, the exact criteria for success varies markedly. Most consider motor results only, with surgical success being ocular alignment of ± 5 to 15 PD [3,6] and success rates being reported as ranging from 42.9% to 88.2% [4,5]. To the best of our knowledge, one-stage surgical outcomes for large-angle IXT have not been reported previously. In this study, we describe the surgical results of one-stage surgery involving either two or three muscles for an IXT with a deviation >60 PD and evaluate risk factors that may affect the success of this surgery. Both the motor and sensory status were used as criteria for assessing final outcomes.

## Patients and Methods

### Patients

The records of 40 IXT patients with exodeviation >60 PD who underwent surgical treatment under general anesthesia in the Eye Hospital, at the Zhongshan Ophthalmic Center, of Sun Yat-Sen University, China, from January 2009 to December 2013 were reviewed retrospectively. The following clinical characteristics were recorded from the patients’ charts: family history, age at onset, age at surgery, best corrected visual acuity, cycloplegic refraction, preoperative motor alignment at distance and near, stereoacuity at distance and near, preoperative ocular motility, presence of an A or V pattern, presence of dissociated vertical deviation (DVD) and surgical methods employed. Patients demonstrating constant exotropia on presentation, any neurologic deficits, coexistent restrictive or paretic strabismus, or previous strabismus surgery were excluded. In this study, we chose records consisting of unilateral lateral rectus recession with medial rectus resection (RR procedure, two-muscle surgery group) for patients with exodeviation ≤70 PD, and bilateral lateral rectus recession with unilateral medial rectus resection for patients with exodeviation >70 PD (three-muscle surgery group). The surgery performed was based on the maximal angle of exotropia as measured preoperatively. All surgeries were performed by four different individuals with expertise in strabismus surgery and all examinations as performed in these patients were conducted by one of the three coauthors. Briefly, ocular alignment was assessed with use of cover/uncover and alternate prism cover testing at distance (6m) in primary and cardinal gaze positions. Motor alignment at near was assessed at 33cm with the use of spectacle correction. Stereoacuity at distance was measured by random-dot stereograms and at near by Titmus stereograms.

### Outcome variables

Orthophoria was considered as being present if the deviation was ≤ 8 PD of exotropia or ≤8 PD of esotropia. Overcorrection was defined as esotropia/phoria >8 PD and undercorrection as exotropia/phoria >8 PD. Stereoacuity < 60 seconds both at near and distance was considered as normal. Both motor and sensory criteria were used in evaluating surgical success rates. Surgical success was considered when the patient was orthophoria with normal stereopsis at near and distance as determined at a minimum of 6 months follow-up. Patients failing to meet the criteria for surgical success were considered as surgical failures (poor outcomes). After patients were categorized into subgroups of surgical success or failure, an attempt was made to define preoperative risk factors for poor outcomes. Factors considered included sex, age at onset, age at surgery, duration of deviation, signs of amblyopia, anisometropia, oblique muscle dysfunction, preoperative stereopsis, preoperative deviation, deviation on the first day after surgery, and surgical methods employed. In addition, considering the importance of surgical age and their ability of rebuilding binocular vision after surgery, we divided patients into three age groups (≤9 years old, >9 and ≤18 years old, and >18 years old) to analyze the surgical result.Written informed consent to participate in this study was obtained from all patients. Institutional approval for this study was obtained from the Research Ethics Board of the Zhongshan Ophthalmic Center, of Sun Yat-sen University, China, and all procedures were performed in accordance with the 1964 Declaration of Helsinki.

### Statistical Analysis

The data were analyzed by SPSS software for Windows (version 17.0, Chicago, IL). Comparison of risk factors involving quantitative data (including age at surgery, age at onset, duration of deviation, prism diopter at distance and near preoperative, anisometropia and deviation at distance on the first day after surgery) were analyzed by the Student’s t-test or Mann-Whitney U-test. The Chi-square test, continuous correction or Fisher exact tests were used for qualitative data (including sex, residual stereopsis, residual fusion, oblique muscle overaction, amblyopia and surgery method). Multivariate logistic regression was used for analyzing the association between risk factors and surgical outcomes. Values presented within the Results section represent the means + SD. A P-Value ≤0.05 was required for results to be considered statistically significant.

## Results

### Clinical Characteristics

A total of 40 records from surgical patients with large-angle IXT were reviewed. Their clinical characteristics are summarized in Table 1. There were 25 patients comprised the two-muscle surgery group and 15 the three-muscle surgery group. The mean preoperative distant and near deviations of the two-muscle group were 67±3PD and 66±20PD, respectively, while that of the three-muscle group were 82±7PD at distance and 90±28PD at near. None of the subjects showed abnormal ocular motility. Neither lateral incomitancy nor normal preoperative stereoacuity were observed in any of the patients.

**Table 1:** Clinical characteristics of patients with large angle IXT.

Mean age at onset	8.9±6.7 years (range: 6 months to 26 years)
Mean duration of deviation	10.3±8.8 years (range: 6 months to 34 years)
Mean age at surgery	18.0±8.1 years (range: 5 years to 37 years)
Male:female ratio	17:23
Family history of strabismus	1/40 (2.5%)
Refractive error	
right eye	-1.87± 2.26D
left eye	-1.44±2.32D
Amblyopia	3/40 (7.5%)
Inferior oblique muscle overaction	7/40 (17.5%)
Dissociated vertical deviation	1/40 (2.5%)
Type of deviation	
basic	34/40 (85%)
pseudo-divergence excess	5/40 (12.5%)
convergence insufficiency	1/40 (2.5%)
Mean preoperative angle of deviation	
Near (33cm)	75±26PD
Distant (6m)	73±9PD
Residual stereoacuity	2/40 (5%)
Surgery approach	
RR	25/40 (62.5%)
Three horizontal muscles	15/40 (37.5%)

### Surgical outcomes and risk factor analysis for poor outcomes

In patients with two-muscle surgery, the mean recession dose was 8.3± 0.7 mm (range: 8–10 mm) and the mean resection amount was 8.1± 0.7 mm (range: 8–9 mm). Of the 15 patients with three-muscle surgery, the mean recession dose was 15.1± 2.3 mm (range: 12–20 mm) for the combined two lateral recti muscles and the mean resection amount was 7.4± 1.3 mm (range: 5–9 mm). Of all the patients, 17 showed mild overcorrection, 16 were orthophoria and 7 showed mild undercorrection on the first day after surgery, with a mean distance esodeviation of 4±8 PD. As determined in their last visit, surgical success (considering only the motor outcome) was achieved in 31 patients (77.5%) with a mean follow-up time of 8.0±4.5 months (range: 6–23 months). Surgical undercorrection was observed in 6 subjects (15%) and overcorrection in 3 cases (7.5%). When patients were classified into surgical success as based on both motor and sensory status, the success rate decreased to 30% (12/40). The success rate for the two-muscle group (32%) was slightly higher than that of the three-muscle group (26.7%), but these differences failed to achieve statistical significance (p = 1.000). An analysis of the preoperative risk factors for surgical failure proved unsuccessful in identifying any significant factors either by univariate or multivariate analysis. In addition, when the patients were divided into three age groups, there were no statistic difference of surgical success among them (Table 2).

**Table 2:** Preoperative risk factors for poor outcomes.

Factors	Success group	Failure group	P-Value	
			Univariate Analysis	Multivariate Analysis
Sex (male/female)	5/7	12/16	0.944^a^	-
Residual stereopsis(n)	1	1	0.515^b^	-
Oblique muscle overaction(n)	2	5	0.654^b^	-
Amblyopia (n)	1	2	0.668^b^	-
Age at surgery	16.2±1.9	18.8±1.6	0.358^c^	-
Age groups (n)			0.263^b^	
≤9 years old	1	5		
> 9 years old and ≤18 years old	7	8		
>18 years old	4	15		
Age at onset	10.8±2.2	8.0±1.2	0.233^c^	-
Duration of deviation	7.8±1.3	11.4±1.6	0.300^d^	-
Deviation at distance (PD)	71±3	73±2	0.671^d^	-
Deviation at near (PD)	73±7	76±5	0.707^c^	-
Anisometropia (D)	-0.37±0.48	-0.46±0.19	0.268^d^	-
Surgical methods (n)			1.000^e^	-
RR	5	20		
Three horizontal muscles	4	11		
Deviation at distance on the first day after surgery (PD)	+6.3±2.8^f^	+2.5±1.4	0.228^d^	-

a Chi-square test.

b Fisher exact tests.

c Independent samples t test.

d Mann-Whitney test.

e continuous correction.

f deviation of esotropia

### Surgical complications

Six patients demonstrated surgical undercorrection with a mean distance exodeviation of 12±3PD. Three of these patients were in the two-muscle group and three in the three-muscle group. Three patients showed a mean distance overcorrection esodeviation of 13±4 PD, with two of these patients being in the two-muscle group and one in the three-muscle group. There were no statistically significant differences between these two groups. All patients with undercorrection were satisfied with their cosmetic results and required no subsequent surgery. One patient in the two-muscle group required a second surgery due to overcorrection with an 18 PD esodeviation as revealed at two months after surgery but achieved a satisfactory result following medial rectus recession of the resected muscle from the first surgery. Seven subjects (17.5%) showed an abduction deficit during the initial postoperative stage, with five of these cases being in the two-muscle group and two in the three-muscle group. There were no statistically significant differences between these two groups. The abduction deficit was rectified in all of these patients as assessed at their final visit. No diplopia was observed in our cohort.

## Discussion

Though surgical and non-surgical management of IXT has been extensively studied, little is known about surgical treatment for large-angle IXT. The currently recommended surgical approaches include one-stage, two-stage, two-muscle, three-muscle and four-muscle procedures [5,7–9]. Most authors stress that, initially, surgery of up to two muscles should be performed in an one-stage surgery and the remaining exodeviation be corrected by a second-stage surgery [7]. Large bilateral lateral rectus recession of 8–14 mm has been reported for large-angle exotropia, showing no symptomatic motor deficit after surgery[10,11]. This is contrary to the opinion that lateral rectus recession should be restricted to 7 mm in cases of an abduction deficit resulting from the surgery [12]. Schwartz et al proposes that a two-muscle surgery was sufficient for large angle exotropia, with 77% of their 22 cases achieving a postoperative alignment of ±15 PD and asymptomatic abduction deficits being observed in only a few cases [10]. Celebi et al reported a 76% success rate of within ±15 PD in 33 cases with an approximately 50–65 PD exodeviation using a bilateral lateral rectus recession of 8.0 to 9.5 mm, and no abduction deficit [11]. Ganguly et al reported a 83.3% success rate in 48 adults with approximately 40–80 PD exodeviation as achieved with unilateral two-muscle surgery [13]. While two-muscle surgery may be an appropriate choice for “medium angle” exotropia, three- or four-muscle procedures may be needed for the very “large-angle” exotropia [14]. Livir-Rallatos stated that in their 63 cases, two-muscle surgery was appropriate for exodeviations up to 50PD with a success rate of 71%, but not for larger than 50PD, where the success rate decreased to 18% [15]. Lau et al reported a success rate of 88.2% with one-stage three-muscle surgery in 24 patients with >60 PD exotropia in their intermittent group and 42.9% in their constant group [5]. Li et al achieved a success rate of 83% using three-muscle surgery in 23 patients with a mean exodeviation of 130 PD [16]. One-stage four-muscle surgery had also been suggested for correcting large-angle exotropia [17,18]. Peters et al reported a success rate of 87.5% within ±10 PD by performing symmetric bilateral medial rectus resections and lateral rectus recessions in 8 patients with a mean exotropia of 63 PD [18].

In none of these studies were surgical results of a single stage procedure for large-angle IXT described. In the present study, we reviewed IXT patients with deviations > 60 PD subjected to one-stage, two- or three-muscle procedures. The definition of “large angle” in our cohort was greater than that typically used by others [3,4,11,15] but similar to that of Lau [5]. Considering that the BLR procedure was more likely to complicate undercorrection than that obtained with RR [19], we selected either the RR procedure or BLR with unilateral medial rectus resection. The mean exodeviation was 73PD at distance in our 40 patients. A large RR procedure was performed in cases with a mean recession of 8.3 mm and resection of 8.1 mm in 25 patients with exodeviation of ≤70 PD and >60 PD. In the remaining 15 cases with exodeviation >70 PD a three-muscle surgery with a mean recession dose of 15.1 mm of the combined two lateral rectus muscles and a mean resection amount of 7.4 mm was performed. Satisfactory motor outcomes were obtained in 77.5% of the patients in our study, which is similar to that reported by others [10,11,15].

As the surgical age of patients with large-angle IXT is typically older, they have less opportunity for restoration of binocular vision than those with mild or moderate-angle IXT. The mean age at surgery in our cases was 18.0 years old, which was substantially older than that of 5–6 year old IXT patients reported in prior studies [20,21]. However, older children and even adults are capable of reconstructing their binocular vision postoperatively [18,22]. Peters reported that in 4 of their 8 adult cases, stereoacuity of approximately 200–40 seconds of arc was obtained postoperatively [18]; and binocularity was restored in 8 of 26 adult patients in a study reported by Currie [22]. When adding a sensory outcome into the criteria for surgical success we found that 12 of the patients in our study (30%) with the mean age of 16 years old at surgery recovered normal stereoacuity as tested both at near and distance. What’s more, patients with different age (≤ 9 years old, > 9 and ≤ 18 years old, and > 18 years old) showed no significant difference in rebuilding their postoperative normal binocular vision in present study.

Previous risk factors associated with poor outcomes have been reported to include preoperative deviation, surgical method and amblyopia [3,9]. Patients with IXT have a tendency toward exodrift postoperatively and the extent of this tendency increases over time [23,24]. Surgical dose for the largest angle of exotropia and a controlled <20 PD overcorrection in the initial postoperative phases are the accepted measures to contain exotropic drift [25]. Based on this criterion, a substantial surgical dose was used to obtain mild overcorrection in the initial stage after surgery in present study. In our study, risk factors for poor surgical results were analyzed using univariate and multivariate analysis. However, none of the factors examined including sex, age at onset, age at surgery, duration of deviation, anisometropia, amblyopia, oblique muscle dysfunction, preoperative stereopsis, preoperative deviation, deviation on the first day after surgery or surgical method employed revealed any statistically significant associations. While the success rate of 32% in the two-muscle group was slightly greater than that of the 26.7% in the three-muscle group, these differences failed to differ statistically. Accordingly, we conclude that either the two- or three-muscle procedure is appropriate for one-stage surgery in large-angle IXT patients, with the selection of either being dependent upon the amount of exodeviation.

In our study, 6 cases (15%) showed surgical undercorrection and 3 cases (7.5%) overcorrection. Although patients in the two-muscle surgery group showed a lower rate of undercorrection (12%) than those of the three-muscle surgery group (20%), these differences were not statistically significant. All cases with undercorrection were satisfied with their postoperative outcomes and did not required further surgery. Rates of overcorrection in both the two- (8%) and three-muscle (6.7%) groups were similar. One patient with an 18 PD overcorrection in the two-muscle group required a subsequent surgery, but achieved a satisfactory result following medial rectus recession of the resected muscle from the first surgery. Seven subjects showed asymmetric abduction deficits during the initial postoperative stages. These deficits were temporary and complete recoveries were obtained when assessed in their final visit, which were similar to that reported by Schwartz and Celebi [10,11]. In our opinion, this temporary limitation of abduction might result from a combination of postoperative eye pain and the large extent of the recession or resection procedure. In this way, the motor component producing this abduction deficit would improve as the pain subsides and normal function would be restored within the recessed muscle.

This study has several limitations that should be noted. It was a retrospective study with a relatively small sample size. The onset of exotropia was based on patient history, which can be subject to recall bias. Patients were not randomly assigned to the two surgery groups. Although relatively uniform surgical procedures and examinations were followed in our hospital, it remains possible that intervening clinical differences exist among the four separate surgeons and three study examiners, Finally, there were variations in the follow-up times among individual cases and the average follow-up period may have been too brief for evaluating the long-term therapeutic outcome.

## Conclusion

Single-stage two- or three-muscle surgery provides an effective means for treatment of large-angle IXT. When both motor and sensory status are included within the criteria for success, success rates decrease. A large amount of lateral rectus recession and medial rectus resection was safe for large-angle IXT, producing no symptomatic postoperative motor deficit.
